# Optimal costs of HIV pre-exposure prophylaxis for men who have sex with men

**DOI:** 10.1371/journal.pone.0178170

**Published:** 2017-06-01

**Authors:** Jennie McKenney, Anders Chen, Karen W. Hoover, Jane Kelly, David Dowdy, Parastu Sharifi, Patrick S. Sullivan, Eli S. Rosenberg

**Affiliations:** 1 Department of Epidemiology, Rollins School of Public Health, Emory University, Atlanta, Georgia, United States of America; 2 Department of Medicine, University of Washington, Seattle, Washington, United States of America; 3 Division of HIV/AIDS Prevention, National Center for HIV/AIDS, Viral Hepatitis, STD, and TB Prevention, CDC, Atlanta, Georgia, United States of America; 4 HIV/AIDS Epidemiology Unit, Georgia Department of Public Health, Atlanta, Georgia, United States of America; 5 Department of Epidemiology, Johns Hopkins Bloomberg School of Public Health, Baltimore, Maryland, United States of America; British Columbia Centre for Excellence in HIV/AIDS, CANADA

## Abstract

**Introduction:**

Men who have sex with men (MSM) are disproportionately affected by HIV due to their increased risk of infection. Oral pre-exposure prophylaxis (PrEP) is a highly effictive HIV-prevention strategy for MSM. Despite evidence of its effectiveness, PrEP uptake in the United States has been slow, in part due to its cost. As jurisdictions and health organizations begin to think about PrEP scale-up, the high cost to society needs to be understood.

**Methods:**

We modified a previously-described decision-analysis model to estimate the cost per quality-adjusted life-year (QALY) gained, over a 1-year duration of PrEP intervention and lifetime time horizon. Using updated parameter estimates, we calculated: 1) the cost per QALY gained, stratified over 4 strata of PrEP cost (a function of both drug cost and provider costs); and 2) PrEP drug cost per year required to fall at or under 4 cost per QALY gained thresholds.

**Results:**

When PrEP drug costs were reduced by 60% (with no sexual disinhibition) to 80% (assuming 25% sexual disinhibition), PrEP was cost-effective (at <$100,000 per QALY averted) in all scenarios of base-case or better adherence, as long as the background HIV prevalence was greater than 10%. For PrEP to be cost saving at base-case adherence/efficacy levels and at a background prevalence of 20%, drug cost would need to be reduced to $8,021 per year with no disinhibition, and to $2,548 with disinhibition.

**Conclusion:**

Results from our analysis suggest that PrEP drug costs need to be reduced in order to be cost-effective across a range of background HIV prevalence. Moreover, our results provide guidance on the pricing of generic emtricitabine/tenofovir disoproxil fumarate, in order to provide those at high risk for HIV an affordable prevention option without financial burden on individuals or jurisdictions scaling-up coverage.

## Introduction

Men who have sex with men (MSM) represent approximately 4% of the United States (U.S.) adult male population, however, they are disproportionately affected by HIV due to their increased risk of infection [1, 2]. In the U.S., MSM account for almost half of people living with HIV and 65% of all new HIV diagnoses [3, 4]. The majority of new diagnoses are in persons of color [3].

Oral pre-exposure prophylaxis (PrEP) can be a highly effective HIV prevention strategy that has the potential to decrease the number of new HIV infections among MSM in the U.S [5, 6]. The original iPrEX landmark study of PrEP in MSM found an overall 44% reduction of HIV transmission. Effectiveness was highly dependent on adherence, with a 92% reduction in the high-adherence subgroup [7]. Subsequent studies have found that PrEP reduced the incidence of HIV infection by as much as 99% if taken daily [8–10]. In 2012 the U.S. Food and Drug Administration approved the use of daily oral tenofovir disoproxil fumarate and emtricitabine (TDF-FTC) for PrEP, and in 2014 the Centers for Disease Control and Prevention (CDC) published clinical guidelines for its use as PrEP to prevent acquisition of HIV infection among high-risk individuals, including MSM, heterosexual men and women, and people who inject drugs [9–11]. Recent mathematical models have demonstrated impact and cost-effectiveness of PrEP among U.S. MSM similar to findings in clinical trials, but have produced mixed results in terms of cost-effectiveness [12–16].

Despite overwhelming evidence of the effectiveness of PrEP at reducing the risk of acquiring an HIV infection, its uptake in the U.S. has been low [17–19]. Uptake has increased markedly over the past few years among white men, yet only a small fraction of the 400,000 men with PrEP indications were prescribed PrEP [20]. Compared to white men, uptake has been very low among black men [21]. In large part, the low uptake can be attributed to cost barriers [17–19, 22– 24]. Out-of-pocket PrEP costs approximately $1500 per month, although manufacturer and state and city medication assistance programs might pay prescription costs for those who qualify. The majority of private insurance companies cover PrEP, but high insurance premiums, deductibles, and co-payments can make PrEP unaffordable for low-income, high-risk individuals. As jurisdictions and health organizations make important decisions about supporting PrEP scale-up, the clinical and economic impact that results from high drug pricing needs to be understood. Previous analyses [12–16] have evaluated PrEP cost-effectiveness at the current drug price, generally concluding that PrEP is cost-effective in high risk populations (e.g. high background HIV prevalence). In the analysis we describe in this article, we consider cost-effectiveness under alternative pricing for PrEP, in order to help identify a price point at which large-scale delivery of PrEP to MSM is economically justified.

## Methods

### Model structure

We modified a previously published and described decision-analysis model [12] to estimate the cost effectiveness ratio (CER), or the cost per quality-adjusted life year (QALY) gained, comparing one year of PrEP to no PrEP over a lifetime horizon, incorporating first-order transmission of HIV. Our primary outcome measures were: 1) the cost per QALY gained; and 2) the PrEP drug cost required to meet specific cost-effectiveness thresholds, as modeled across a number of different scenarios.

### Updated parameter estimates

We updated three parameter values for this analysis. The per-act probability of HIV acquisition for receptive anal intercourse and the risk ratio for insertive intercourse were changed to reflect a recent meta-analysis [25]. Prior estimates remain referenced [26–28]. Additionally, the lifetime cost of HIV per patient was updated to reflect current estimates of the costs associated with an HIV diagnosis [16, 29, 30]. The full parameters can be found in Table 1.

**Table 1:** Parameter values for cost-effectiveness analysis.

Parameter	Value	Sensitivity Range	Reference
**Risk of HIV acquisition**			
**Probability of HIV acquisition per sex act with HIV + partner**^a^	0.0138	0.0102–0.0186	[25]
**Insertive anal sex act with HIV + person (risk ratio)**	0.08	0.03–0.2	[25]
**HSV2 seropositive (risk ratio)**	2.14	1.5–3	[31]
**HSV2 prevalence**	0.196	0.05–0.4	[32]
**ART (risk ratio)**	0.09	0.05–0.2	[27], [33],[34]
**ART prevalence**^b^	0.36	0.2–0.6	[35]
**PrEP effectiveness (risk ratio)**	0.56	0.37–0.85	[7]
**Condom use (risk ratio)**	0.2	0.1–0.3	[36]
**Condom use (prevalence)**	0.4	0.2–0.6	[16]
**Untreated GC/CT/Syphilis (risk ratio)**	3.5	2–5	[37]
Untreated GC/CT/Syphilis (prevalence)*	0.11	0.05–0.4	[38]
**Average number of sex acts per month**	7.06	5–10	[39]
**HIV prevalence, MSM age 13–64**	0.19	0.05–0.4	[40]
**Costs, 2015 US$**			
**Annual cost of PrEP**^c^	10,711	4,772–15,000	[16]
**Lifetime cost per HIV patient, discounted**	327,503	150,000–500,000	[16], [29], [30]
**Average cost per case of STI treated (men)**^d^	180	99–295	[38], [41], [42]
**Average cost per STI test**	67	27–80	[16]
**QALYs**			
**QALY gained per case of HIV averted, discounted**^e^	2.24	1.07–3.2	[16], [41]
**QALY lost per additional STI**^f^	0.02	0.01–0.03	[29], [42–45]

^a^ Risk per unprotected receptive anal sex act, with no ART use by the infected partner, and no PrEP use.

^b^ Prevalence of prescribed ART

^c^ Drug cost: $10,711, physician visits: $345, renal function tests: $15, HIV tests: $27.

^d^ Cost per case of GC/CT/syphilis treated: $79/30/709; relative proportion of GC/CT/syphilis: 0.45/0.353/0.194.

^e^ Disability weight for asymptomatic HIV/symptomatic HIV/AIDS: 0.94/0.82/0.7; years lived per stage of HIV infection: asymptomatic: 7, symptomatic: 21, AIDS: 7.

^f^ Disability weight for symptomatic GC or CT/GC or CT epididymitis: 0.933/0.833; prevalence of symptomatic GC/CT: 0.31/0.28; prevalence of epididymitis: 0.0069/0.0093; disability weights for primary/secondary/tertiary syphilis: 0.985/0.952/0.717; prevalence of primary or secondary syphilis/tertiary syphilis: 0.61/0.009; years disability with tertiary syphilis: 5.

* Abbreviations: GC: Gonorrhea, CT: chlamydia trachomatis; QALY: quality-adjusted life-year.

### HIV acquisition and disease progression

HIV acquisition was modeled as an independent risk per unprotected receptive anal intercourse act with an HIV-infected partner. To this risk we applied literature-estimated risks and prevalence of several modifiable variables, as per Table 1 [7, 16, 25, 27, 29–45]. We then used estimates of HIV prevalence among members of a person’s sexual network to calculate a per-act risk of HIV acquisition. HIV disease progression is then modeled linearly as per previous cost-effectiveness analyses, using literature estimates of life expectancy, stages of HIV disease progression and duration in each stage (with and without treatment), disability-adjusted weights associated with each HIV disease stage, and cost of HIV care. Behavioral disinhibition was modeled in some scenarios, as an increase in sex acts and decrease in condom use, with associated increase in HIV transmission and STI transmission, and resulting changes in both QALYs and costs. Additional detail has been previously described [12].

### Economic analysis

#### Cost of PrEP

The cost of PrEP and the cost per QALY gained were estimated taking a societal perspective and using a lifetime analytic horizon [16, 46]. When implemented according to CDC clinical guidelines, the costs associated with PrEP include HIV screening before initiation of PrEP; quarterly HIV screening after introduction; renal function tests before initiation, and bi-annually thereafter; bi-annual testing for sexually transmitted infections (STIs); and drug and physician costs. QALYs associated with PrEP use were estimated using literature estimates of life expectancy and quality of life of a HIV-infected individual according to stage of infection [16]. Toxicity and drug resistance due to PrEP were not included in the model.

#### Drug price and cost per QALY threshold analysis

We calculated the cost per QALY gained for 4 strata of overall PrEP cost (a function of both drug cost and provider costs): $11,884, $7,600 (40% reduction in PrEP drug cost), $5,457 (60% reduction in PrEP drug cost), and $3,315 (80% reduction in PrEP drug cost), while varying background HIV prevalence (10%, 20%, 30%, and 40%), behavioral disinhibition (no disinhibition and 25% increase in sexual risk, including a 25% decrease in condom use, 25% increase in sexual encounters, and 25% increase in STIs), and PrEP adherence/efficacy levels (low adherence/32% risk reduction, base-case /44% risk reduction, and high adherence/92% risk reduction). Percent drug cost reductions were selected *a priori* and were based on a report published by the Health and Human Services (HHS) Office of the Assistant Secretary for Planning and Evaluation (ASPE), examining literature estimates of generic drug prices and the effect of market saturation on generic drug prices [47]. We also estimated the requisite PrEP drug cost in order to fall at or under 4 strata of cost per QALY thresholds: Cost saving, $50,000/QALY, $100,000/QALY, and $150,000/QALY, while varying the same parameters listed above (HIV prevalence, PrEP effectiveness and behavioral disinhibition). Cost per QALY thresholds were determined *a priori* and were based on literature estimates of acceptable cost per QALY thresholds [2, 48].

### Sensitivity analysis

The previously published paper introducing this model includes a one-way sensitivity analysis of all parameters which impacted the CER significantly. We include an updated one way sensitivity analysis based on the updated model input parameters. We also conducted a probabilistic uncertainty analysis on the primary outcome measures, using point estimates and upper and lower bounds estimated from the literature (Table 1). We used Latin Hypercube Sampling to select values from beta distributions with shape parameter (alpha) = 4 and minimum/maximum value as given in Table 1. We then calculated 95% uncertainty ranges as the boundaries of the 2.5^th^ and 97.5^th^ percentile from 10,000 simulations.

## Results

With updated parameter estimates, the cost per QALY gained for our base case scenario is estimated at $64,000 [95% UR: cost saving to $420,000]. The original analysis [12] estimated a cost per QALY gained of $160,000 (95% UR: cost saving to $740,000). The difference in the CER reflects the updated parameter estimate of the per-act probability of HIV acquisition for receptive anal intercourse, per a 2014 meta-analysis [25], which presents a significantly higher per-sex act transmission risk, thereby improving the absolute risk reduction and cost effectiveness of PrEP.

**Fig 1:**
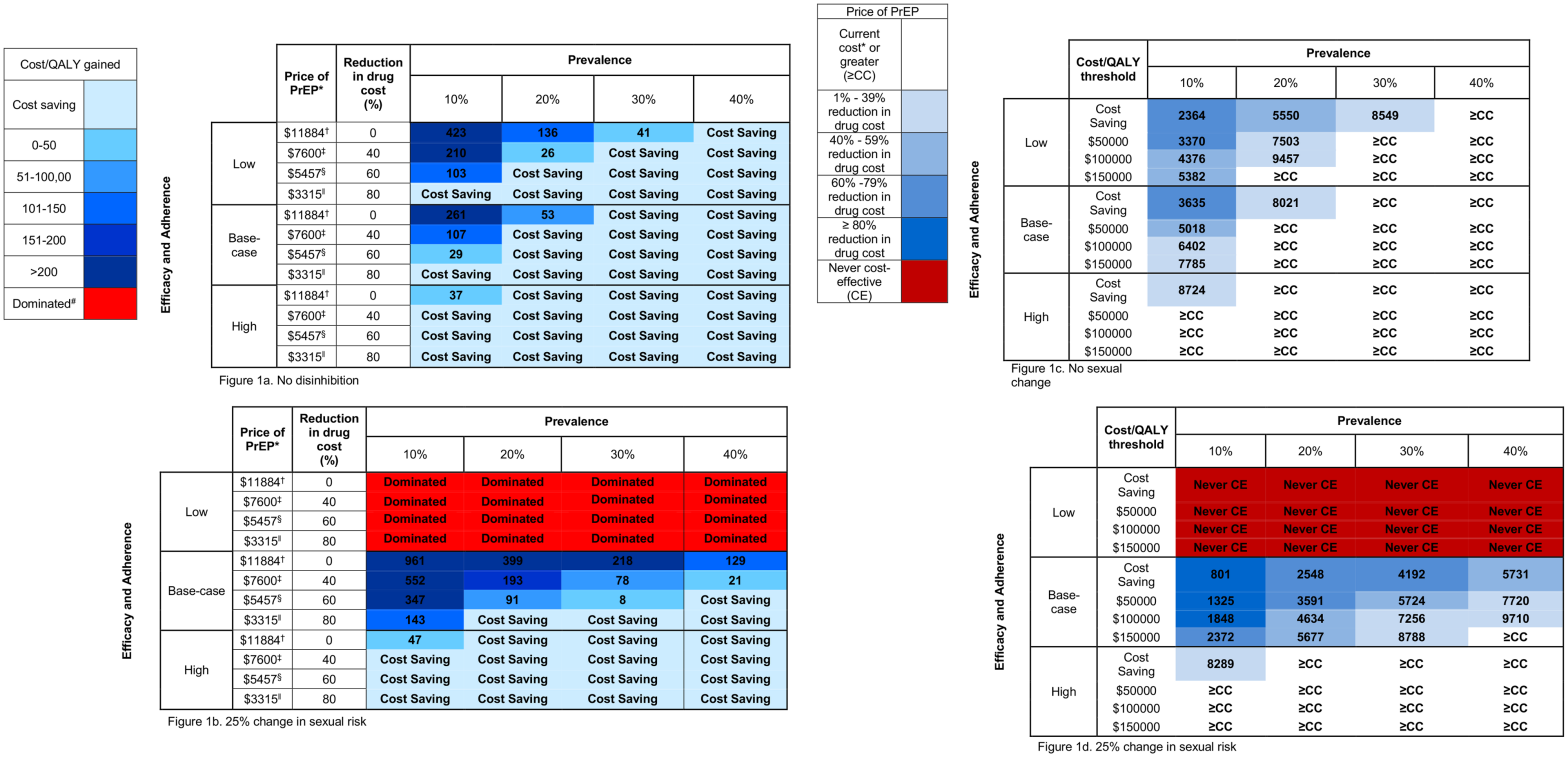
**a-b: Incremental cost-effectiveness of PrEP (cost per QALY gained in thousands of US$)** *Combines drug cost, physician visits, and laboratory testing: drug cost: $10711/year, cost of physician visits: $1035/year, cost of renal function test: $30/year, cost of HIV testing: $108/year ^†^Drug cost: $10711/year ^‡^Drug cost: $6427/year ^§D^rug cost: $4284/year ^‖^Drug cost: $2142/year ^#^Dominated refers to cost-effectiveness scenarios with higher cost and worse outcomes. c-d: **PrEP drug cost stratified by cost per QALY threshold.** *Current drug cost estimated at $10711.

Fig 1 displays additional results from the analysis. Fig 1a and 1b estimate the cost per QALY gained stratified by four PrEP price points, while varying 3 different population parameters: background HIV prevalence, PrEP adherence/efficacy, and change in sexual behavior. Fig 1a shows scenarios without sexual disinhibition. When PrEP drug costs were reduced by 60%, PrEP was cost saving over the range of adherence/efficacy levels, at a background HIV prevalence of 20% or greater. PrEP becomes cost saving when drug cost was reduced by 80%, over the entire range of both adherence/efficacy and background HIV prevalence.

Fig 1b shows scenarios when sexual disinhibition is present. At low adherence/efficacy, PrEP is always dominated regardless of PrEP drug price (e.g. total QALYs lost due to sexual disinhibition are greater than QALYs gained due to PrEP, so price is irrelevant) At base case PrEP efficacy levels, even with 60 or 80% reduction in drug costs, PrEP is not cost effective at the $100,000 per QALY threshold at lower HIV prevalence levels (10%), but is cost effective or cost saving at higher HIV prevalence (20% or greater). With high adherence/efficacy, at a 40% price reduction or greater PrEP is cost saving across all HIV prevalence levels.

Fig 1c and 1d estimate what the PrEP drug price would need to be set at in order to fall at or under four different cost per QALY thresholds, when varying the same 3 population parameters listed above. Without reducing PrEP drug cost, PrEP is already cost-effective (and under some scenarios cost saving) when PrEP adherence/efficacy is high. In other scenarios, PrEP would need to undergo a price reduction to be cost effective (at the $100,000/QALY threshold) or cost saving.

With base case adherence/efficacy and no disinhibition, in order for PrEP to be cost saving, drug cost would need to be reduced to $3,635 [95% UR: $1279-$7967] (66% reduction) at the lowest background HIV prevalence and to $8,021 [95% UR: $3460- no price change] (25% reduction) at a background prevalence of 20%. Under those same conditions, in order for PrEP to be cost-effective at less than $100,000 per QALY gained, drug cost would need to be reduced to $6,402 [95% UR: $2788- no price change] (40% reduction) at a background prevalence of 10%, and at a background prevalence of 20% or greater, PrEP would not need to undergo a price reduction [95% UR: $6421- no price change].

Additionally, for PrEP to be cost saving at base case adherence/efficacy when assuming sexual disinhibition is present, drug cost would be need to be reduced to $801 [95% UR: $0-$3925] (93% reduction) at the lowest background HIV prevalence and to $2,548 [95% UR: $0-$8490] (76% reduction) at a background prevalence of 20%. To be cost-effective at less than $100,000 per QALY gained under those same conditions, drug cost would need to be reduced to $1,848 [95% UR: $0-$6770] (83% reduction) at a background prevalence of 10% and to $4,634 [95% UR: $0- no price change] (57% reduction) at a background HIV prevalence of 20%.

Under scenarios where PrEP adherence/efficacy is low without disinhibition, drug cost would need to be reduced even further to meet cost effectiveness or cost savings threshold, as per Fig 1c. Under scenarios in which PrEP adherence/efficacy was low and disinhibition present, PrEP was never cost-effective.

**Fig 2:**
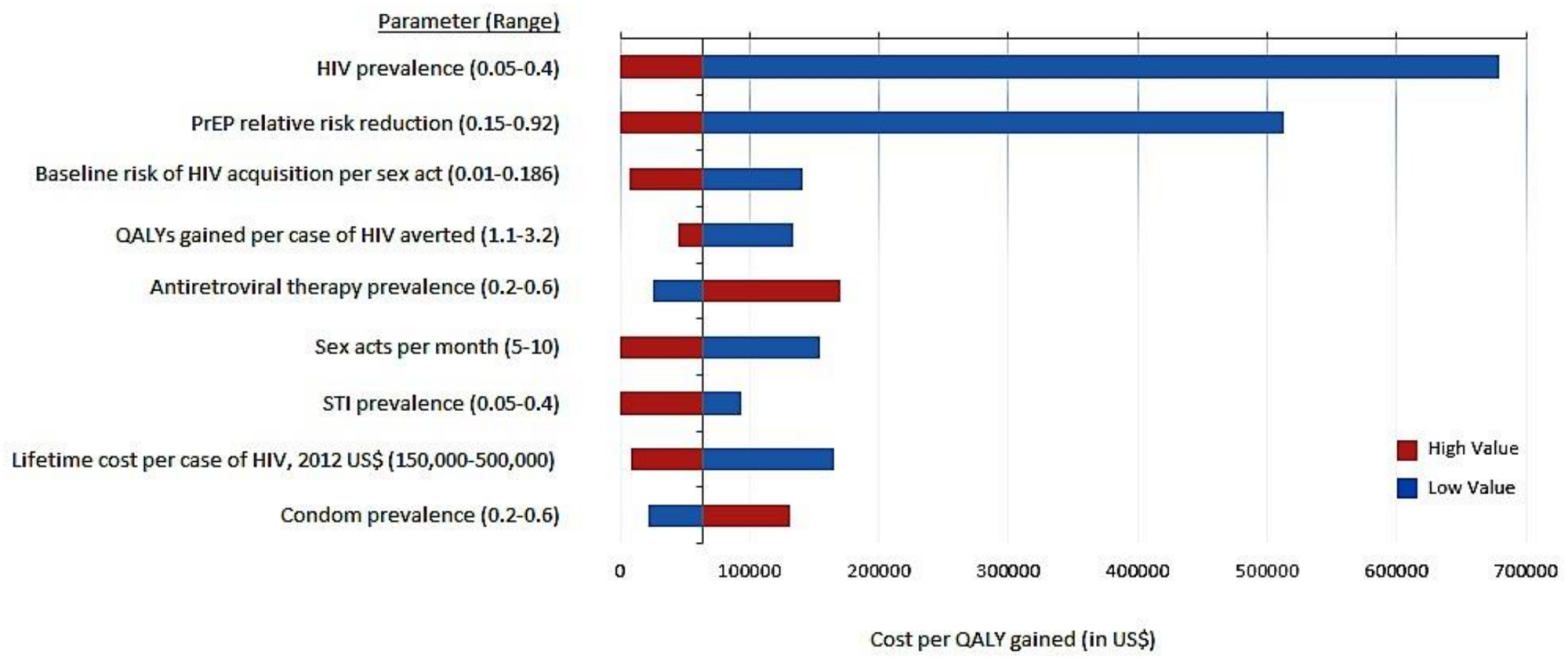
One-way sensitivity analysis of PrEP cost-effectiveness (in US$). The black vertical line represents the base case-scenario relative to no PrEP ($64,000 per QALY gained). Blue bars represent the low value of the range, and red bars represent the high value of the range. Bars to the left of the base case scenario represent more favorable scenarios. Only parameters which affected the cost-effectiveness ratio by more than 50% in either direction are shown.

Fig 2 shows the updated one-way sensitivity analysis of key model parameters. Results from the tornado plot suggest that HIV prevalence and PrEP relative risk reduction/adherence are the two parameters that affect the cost-effectiveness of PrEP the most. Additionally, the estimated risk of HIV transmission per sex act clearly has an important impact, as the change in this parameter value in our current analysis leads to a base case cost per QALY gained of $64,000 compared to the original analysis of $160,000 [12]. Together, this reflects that PrEP cost effectiveness in the real world varies tremendously based on both biologic factors and patient/population specific factors. Table 2 shows QALYs gained, cost, and cost-effectiveness of PrEP vs. No Prep under certain scenarios selected.

**Table 2:** Accumulated quality-adjusted life years gained, cost, and cost-effectiveness of selected scenarios.

Treatment scenario	Effectiveness, QALYs gained	Cost, relative to no PrEP (US dollars)	Cost Effectiveness (cost per QALY gained) compared to no PrEP
**Low adherence, low HIV prevalence**	4.02	1,700,000	423,000
**Low adherence, low HIV prevalence, 60% drug cost reduction**	4.02	415,000	103,000
**Moderate adherence, low HIV prevalence**	5.53	1,446,000	261,000
**Moderate adherence, low HIV prevalence, 60% drug cost reduction**	5.53	161,000	29,000
**Moderate adherence, moderate HIV prevalence (base case)**	10.24	654,000	64,000
**Moderate adherence, moderate HIV prevalence, 40% drug price reduction**	10.24	-203,000	cost saving
**High adherence, low HIV prevalence**	11.6	428,000	37,000
**High adherence, low HIV prevalence, 40% reduction in drug cost,**	11.6	-429,000	cost saving

* We do not include incremental cost effectiveness ratios (ICER): as this model represents different scenarios of the same intervention, comparing ICERs would lead to all scenarios dominated except the one with lowest drug cost and highest PrEP effectiveness.

## Discussion

Our analysis found that at base-case levels of adherence and efficacy, PrEP would be cost-effective over a range of higher background HIV prevalence. In some cases, PrEP would be cost-saving, if drug costs were reduced. For example, PrEP would be cost-effective if the drug price was reduced by at least 60% and used by MSM with base-case level of PrEP adherence and efficacy, who practice disinhibition, and live in a population with a HIV prevalence of 10% or greater, representing a realistic setting. Based on published data [49, 50], 34 U.S. jurisdictions have populations of MSM with an HIV prevalence of 10% or greater. This setting also reflects adherence and efficacy levels found in the iPrEx clinical trial, and accounts for decreased condom use with PrEP, although evidence for risk compensation is mixed [6, 9, 10, 51–54]. In scenarios where adherence and efficacy were low and disinhibition occurred, PrEP would never be cost-effective, highlighting the importance of both behavioral risk reduction interventions for the target population and the need for lower PrEP pricing.

A reduction in the price of PrEP would benefit a wide range of U.S. MSM, and would make PrEP cost-effective or even cost-saving. Moreover, lower drug costs would likely lead to greater uptake and adherence, and potentially decreased HIV incidence, and thus, decreased HIV prevalence in the population in the future. At its current cost, PrEP is not a cost-effective prevention intervention for all MSM in the U.S [14, 16] However, with reduced PrEP costs we estimated that the cost per QALY gained would be substantially lower than other public health prevention interventions. If PrEP costs were reduced by 80% in the context of base-case level of adherence and efficacy, disinhibition, and a background prevalence of HIV greater than 10%, PrEP would be cost-saving. Hemodialysis for end-stage renal disease is often used as a benchmark for cost per QALY thresholds, at $125,000 to $150,000 per QALY [55–57]. Further, the cost per QALY gained for cervical cancer screening is estimated to be $19,530, higher than the cost per QALY for PrEP when drug cost is reduced by 80% [58]. A recent modeling study of PrEP implementation in England found that the price of PrEP would need to be reduced by 80% or more of its current cost for it to be cost-effective and affordable to scale-up for population impact [59].

Although evidence from clinical trials, demonstration projects, and mathematical models suggest that PrEP could have substantial impact on the HIV epidemic within the U.S., scale-up has been slow. Several barriers have hindered its implementation, including access to health insurance [17–19, 22–24, 60, 61]. Although the Affordable Care Act has reduced the number of Americans without health insurance from 18% in 2013 to 11.9% in 2015, healthcare disparities remain, especially among black Americans who are also disproportionately affected by HIV [60, 61]. A study measuring the PrEP care continuum of MSM in Atlanta, found black MSM were less likely than white MSM to have access to healthcare (53% and 76%, respectively) and to ultimately receive a prescription for PrEP, resulting in only 12.3% of black MSM achieving protection from PrEP, compared to 17.8% of white MSM [60]. Similar results are seen from a meta-analysis examining disparities between black and white MSM that identified structural barriers, such as access to healthcare, as one of the highest ranked disparities affecting HIV acquisition between these two groups [62].

Another barrier to PrEP implementation is the high cost of PrEP medication [17–19, 22–24]. Despite the fact that most private insurance and Medicaid plans include PrEP as a benefit, and medication assistance programs help to pay for PrEP drugs for those who qualify, PrEP is too expensive for many persons who might benefit from it. Out-of-pocket costs hinder the ability of PrEP scale-up for low-income, high-risk individuals, with often unaffordable insurance premiums, deductibles, and co-payments for those with insurance and approximately $1500 per month in drug cost for those without insurance. The availability of a generic form of the drug might help to make PrEP more affordable. Generic drugs are, on average, priced at 80% of the price of brand name drugs [47]. Truvada, the only drug approved for PrEP in the U.S., is protected by a patent that will expire in 2017. Assuming the patent terms of Truvada are not extended, generic formulations will likely be produced and result in reductions in price. However, lower drug pricing typically lags following expiration of a patent and the loss of market exclusivity [63,64]. Market saturation with a generic drug and reduction in the price might not occur immediately or until multiple competitors enter the market [47]. Only two known Abbreviated New Drug Applications (ANDAs) for generic PrEP drugs have been filed by competitors [65, 66], so it is likely that saturation of the PrEP market will be delayed. To facilitate PrEP scale-up for an estimated 1.2 million adults eligible for PrEP [67], the pricing of generic PrEP would need to be substantially less expensive than Truvada.

There are several limitations associated with our study. First, the model does not incorporate the non-linear dynamics of HIV if PrEP were to prevent not only an index infection but also forward transmission of HIV infection in the population. This may lead to a more conservative estimate of the cost effectiveness of PrEP. Second, important heterogeneities are not incorporated in the model. For example, we did not account for dense, associative, and heterogeneous sexual networks that can result in pockets of very high transmission density. These high risk pockets are where PrEP can be most effective and cost effective so the simplification of sexual transmission in our model may again lead to a more conservative estimate of the cost effectiveness of PrEP.

It is important for federal, state, and local policy makers to understand how the cost of PrEP medications might impact their capacity to scale-up it up for MSM. Lower pricing of PrEP drugs ensures that drug formularies include these medications in their top tiers not only for HIV treatment but also for PrEP, and that PrEP medications are included on state Medicaid Preferred Drug Lists. Also, many MSM who would benefit from PrEP do not have private insurance or live in states where Medicaid has not been expanded and lower medication pricing would make self-pay of PrEP more affordable. In addition, with lower drug pricing, state and local PrEP assistance programs, such as the Washington State PrEP Drug Assistance Program, would be able to expand PrEP services to an increased number of men [68].

Jurisdictional decisions on HIV prevention and treatment are constrained by limited resources, and ensuring access to PrEP for eligible high-risk persons is a high priority [69]. The cost-effectiveness of a public health intervention is often considered by funders and legislators, and enumerating threshold conditions at which PrEP becomes cost-saving provides guidance for the direction of funds. Results from this analysis provide the tools needed to make sense of cost-breaks and guide public health decision-making. For example, Georgia has a high HIV prevalence, so PrEP is cost-effective at a higher price break than in a lower prevalence state such as Montana, where PrEP scale-up might not be feasible until prices are substantially lower.

PrEP has the potential to reduce HIV incidence in the U.S. Our analysis suggests that, for many scenarios, PrEP is cost-effective compared to traditional thresholds often used to benchmark cost-effectiveness. If the price of PrEP were lowered by its manufacturer or with eventual generic manufacturing, PrEP could be cost-saving in most jurisdictions. Our results provide support for continued efforts to implement PrEP for MSM in the U.S., and suggest that with medication price reductions PrEP could be accessible to those at highest risk of acquiring HIV but without personal or societal financial burden.
