# Correction: Optimal costs of HIV pre-exposure prophylaxis for men who have sex with men

**DOI:** 10.1371/journal.pone.0182593

**Published:** 2017-07-27

**Authors:** Jennie McKenney, Anders Chen, Karen W. Hoover, Jane Kelly, David Dowdy, Parastu Kasaie, Patrick S. Sullivan, Eli S. Rosenberg

The sixth author’s name is incorrect. The correct name is Parastu Kasaie. The correct citation is McKenney J, Chen A, Hoover KW, Kelly J, Dowdy D, Kasaie P, et al. (2017) Optimal costs of HIV pre-exposure prophylaxis for men who have sex with men. PLoS ONE 12(6): e0178170. https://doi.org/10.1371/journal.pone.0178170.
